# Promoting healthspan and lifespan with caloric restriction in primates

**DOI:** 10.1038/s42003-019-0348-z

**Published:** 2019-03-15

**Authors:** Fabien Pifferi, Jérémy Terrien, Martine Perret, Jacques Epelbaum, Stéphane Blanc, Jean-Luc Picq, Marc Dhenain, Fabienne Aujard

**Affiliations:** 1 UMR CNRS/MNHN 7179, Mécanismes Adaptatifs et Evolution, 1 Avenue du Petit Château, 91800 Brunoy, France; 2 Unité Mixte de Recherche en Santé 894 INSERM, Centre de Psychiatrie et Neurosciences, Université Paris Descartes, Sorbonne Paris Cité, 75014 Paris, France; 3 Université de Strasbourg, IPHC, 23 rue Becquerel, 67087 Strasbourg, France; 4 CNRS, UMR7178, 67087 Strasbourg, France; 5 Laboratoire de psychopathologie et de neuropsychologie, E.A. 2027, Université Paris 8, 2 rue de la liberté, 93000 St. Denis, France; 6 CNRS, CEA, Université Paris-Sud, Université Paris-Saclay UMR 9199, Neurodegenerative Diseases Laboratory, 92265 Fontenay-aux-Roses, France; 7 Commissariat à l’Energie Atomique et aux Energies Alternatives (CEA), Direction de la Recherche Fondamentale (DRF), Molecular Imaging Research Center (MIRCen), 92265 Fontenay-aux-Roses, France

## Abstract

Recent data confirmed the efficiency of caloric restriction for promoting both healthspan and lifespan in primates, but also revealed potential adverse effects at the central level. This paper proposes perspectives and future directions to counterbalance potential adverse effects. Efforts should be made in combining nutrition-based clinical protocols with therapeutic and/or behavioral interventions to aim for synergetic effects, and therefore delay the onset of age-related diseases without adverse effects.

## Abstract (web summary)

Fabien Pifferi et al. discuss the latest research in using caloric restriction for promoting healthspan and lifespan in primates. Their Comment touches on their previous study, addressing how to combine nutrition-based clinical protocols with interventions to delay the onset of age-related diseases.

Caloric restriction (CR) is a nutritional intervention consisting in eating less without malnutrition. Its beneficial effects on healthspan and lifespan are very clear in short-lived species (both invertebrates and vertebrates) while they have long been debated in primates. In 2006 was initiated a long-term project, called the “Restrikal study”, which was aimed at assessing the effects of chronic-moderate CR in a nonhuman primate species^1^. Our 2018 publication of the main results of the Restrikal study^2^ brought new arguments in this debate.

In this study, we subjected a cohort of young adult gray mouse lemurs to a 30% CR diet, compared to a control group, until their natural death. The gray mouse lemur (*Microcebus murinus*) is a small malagasy primate with numerous similarities to humans, in particular during aging, for which it represents a promising and emerging model^3,4^. Compared to control animals showing a median survival time of 6.4 years, CR extended survival by 50%, reduced aging-associated diseases and preserved loss of brain white matter in several brain regions. However, CR accelerated loss of gray matter throughout much of the cerebrum. Up to five years of treatment, CR did not change the cognitive status in spatial and working memory tests or neuromuscular performances. Thus, chronic-moderate CR does extend lifespan and enhance health of a primate, but at the expense of brain gray matter integrity.

Two studies on the impact of CR on health and lifespan had been previously initiated in rhesus monkeys^5,6^. Their investigators initially reported contradictory results on survival^7^, but finally concluded to a positive impact of CR on health and survival though their mortality curves were still incomplete^8^. In this context, our study brought the most advanced survival dataset for a primate under CR (all control animals were dead at the time of the publication) with very strong effect of calorie restriction on all-cause mortality. Since our publication in *Communications Biology* in April 2018, all calorie-restricted mouse lemurs are now dead, demonstrating an increase in maximum lifespan of more than 22% (13.8 years in the restricted group vs. 11.3 years in the control group, Fig. 1).

**Fig. 1:**
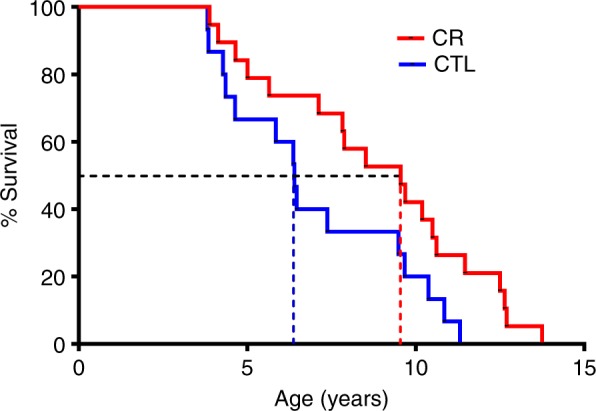
Kaplan–Meier survival curves for overall mortality of mouse lemurs from the control group (CTL—blue) and the calorie-restricted group (CR—red) (*p* log rank = 0.02). The median survival is 6.4 years in control animals and 9.6 years in calorie-restricted animals (dotted lines)

Concomitantly to the publication of our study in mouse lemurs, a conclusive study on the effects of chronic-moderate calorie restriction in humans was issued in cell metabolism^9^. In this study, voluntary subjects were submitted for 2 years to a 15% CR without modifying meals composition. The study found a mean weight loss of 9 kg in the test subjects, while control subjects (who did not modify their dietary habits) did not lose weight. CR in humans induced a slowing in metabolism and decreased production of free radicals. According to the “Rate-of-living Theory of Aging”, metabolic activity is inversely correlated to longevity in mammalians^9^. The “Free Radical Theory of Aging” posits that free radicals production is responsible for the multiplication of cellular lesions leading to aging symptoms^9^. Thus according to these two theories, the changes induced by CR in humans should slow down aging processes and may have visible benefits on clinical outcomes.

## Perspectives and future directions

All studies in primates including those in mouse lemurs, macaques, or humans seem nevertheless to lead to the same conclusion: eating too much is deleterious for health and longevity. However, it remains difficult to determine what is the optimal level of calories for a given species, at a given age. In the Restrikal project, even if reducing the number of calories was strongly efficient to promote health and longevity, it was accompanied by a deleterious effect on brain gray matter integrity. This suggests that, as for most therapies an optimal balance between positive and negative effects should be further studied and biological mechanisms leading to positive and negative effects should be explored. With regards to our data, it seems critical to evaluate the impact of calorie restriction on brain health. Such analyses are merely described in the literature and should be the object of further dedicated studies. The tissues and longitudinal data stored over the course of our 10-year study (see ref. ^1^ for the full description of the project and list of analyses performed) present a unique resource to identify the pathways involved in CR effects in primates in order to optimize the development of nutrition-based clinical interventions to offset age-related morbidity. In addition, the strength to work in lemurs is that, due to the small size of the animals and their relatively short lifespan, it is possible to perform studies involving large cohorts leading to robust results.

One perspective to the Restrikal study would be to develop combined strategies to counterbalance the adverse effects of chronic-moderate CR. One option could be the combination of CR with therapeutic intervention. For example, although still debated^10,11^, resveratrol, which has long been considered as a CR mimetic compound^12,13^, seems to have beneficial effects on health parameters and potentially lifespan, with positive effects on the development of neurodegenerative diseases^14^. Since long-term CR is very difficult to implement in humans because of social and practical constraints, it is of major interest to find compounds that could contribute to slow down the aging process and delay the onset of age-related diseases without modifying, or only slightly modifying, feeding habits. Therefore, it would be very interesting to verify whether resveratrol supplementation induces beneficial effects in mouse lemurs, and whether combined with very moderate (~15%) CR, this treatment could decelerate the brain gray matter atrophy. One additional option would be the improvement of behavioral habits, for example physical activity. Indeed, moderate chronic physical activity is known to enhance cognitive functions and favor neurogenesis^15,16^. Thus, efforts should be put in the future into the investigation of combining very moderate (~15%) CR with daily physical activity (~800 m of walking per animal per day, corresponding at 0.2 m s^−1^).

The question of whether there is a limit for the maximum of human lifespan was recently asked and is strongly debated^17–19^. Nevertheless, the aim of nutrition-based clinical interventions is not necessarily to extend human lifespan, but rather to delay the onset of age-related diseases that are part of the aging process. In this perspective, the most promising option is probably to combine strategies to aim for an optimum of synergic effects between nutritional, behavioral and therapeutic interventions.

## Data Availability

The data corresponding to Figure 1 are available from the authors upon reasonable request.
